# Pediatric Ventriculoperitoneal (VP) Shunt Catheter Migration Into the Pulmonary Trunk: A Case Report and Literature Review

**DOI:** 10.7759/cureus.77088

**Published:** 2025-01-07

**Authors:** Maria Kolcheva, Elvira Emi Abdullaeva, Anders Robert Hakimovich, Eduardo Patricio Pucha Caraguay, Boris Igorevich Oleinikov, Gennady Chmutin, Gervith Reyes Soto, Jose de Jesus Gutierrez Baños, Carlos Castillo-Rangel, Andreina Rosario Rosario, Carlos Ernesto López Lara, Manuel de Jesus Encarnacion Ramirez

**Affiliations:** 1 Neurosurgery, Peoples' Friendship University of Russia, Moscow, RUS; 2 Neurosurgical Oncology, Mexico National Cancer Institute, Tlalpan, MEX; 3 Endovascular Neurosurgery, Hospital Regional 1ro de Octubre Instituto de Seguridad y Servicios Sociales de los Trabajadores del Estado (ISSTE), Mexico City, MEX; 4 Neurosurgery, Hospital Regional 1ro de Octubre Instituto de Seguridad y Servicios Sociales de los Trabajadores del Estado (ISSTE), Mexico City, MEX; 5 Medicine, Autonomous University of Santo Domingo (UASD), Santo Domingo, DOM; 6 Neurosurgery, National Research Mordovia State University, Saransk, RUS; 7 Human Anatomy and Histology, The N.V. Sklifosovsky Institute of Clinical Medicine (ICM), Moscow, RUS

**Keywords:** catheter, hydrocephalus, migration of peritoneal, pulmonary trunk, ventriculoperitoneal

## Abstract

Ventriculoperitoneal (VP) shunting is commonly used to treat hydrocephalus, especially in pediatric patients. Despite its effectiveness, rare complications, like the migration of the distal catheter into the pulmonary trunk (PT), can occur. This case study presents a 17-year-old boy who experienced this complication, highlighting diagnostic challenges, surgical intervention, and outcomes. A review of the literature emphasizes the rarity and clinical management of such cases. A retrospective case review was conducted on a 17-year-old patient with VP shunt catheter migration into the PT. The case was managed at the Morozovskaya City Clinical Hospital, with diagnostic imaging and surgical intervention. A literature review of 25 reported cases from 1993 to 2024 was performed, focusing on demographics, clinical presentations, management strategies, and outcomes. In this case, computed tomography (CT) revealed the distal VP shunt catheter had migrated into the PT. Surgical removal via manual traction was successful, without intraoperative complications. The literature review identified 25 similar cases, most occurring in adults. Clinical presentations varied, with 25.93% being asymptomatic. Surgical outcomes were generally favorable, with few postoperative complications. Accurate diagnosis, typically via CT, is crucial for appropriate management. Surgical removal, often by manual traction under fluoroscopic guidance, is the most effective treatment. Although complications such as arrhythmias or thromboembolism can occur, timely intervention generally leads to positive outcomes, as demonstrated in this case and the literature review.

## Introduction

Ventriculoperitoneal (VP) shunting is a widely used neurosurgical intervention for treating hydrocephalus, particularly in pediatric patients. This procedure effectively reduces intracranial pressure by diverting excess cerebrospinal fluid (CSF) from the brain's ventricles into the peritoneal cavity. Despite its efficacy, VP shunting carries a risk of various complications, including mechanical failures and infections. One of the rarest and most serious complications is the migration of the distal catheter into the pulmonary trunk (PT), as seen in this clinical case of a 17-year-old male patient. This introduction discusses the significance of such complications, explores the mechanisms of catheter migration, and reviews the management strategies for these complex cases.

Hydrocephalus is among the most frequent reasons for pediatric neurosurgery. Countries such as Brazil, the USA, and Canada perform over 40,000 operations annually to address hydrocephalus in children [1,2]. The VP shunt is the gold-standard treatment, consisting of four key components: the proximal catheter, valve, reservoir, and distal catheter. These components work together to drain excess CSF from the ventricles into the peritoneal cavity [3,4]. However, despite their common use, VP shunts are prone to complications. Studies show that up to 23% of children with VP shunts experience shunt dysfunction within the first year [5].

The primary complications of VP shunting include underdrainage, overdrainage, infection, and mechanical failure. As many as 22% of patients will require shunt revision, with mechanical failure being the most frequent reason [6]. One of the rare but documented mechanical failures is the migration of the distal catheter. Migration can occur in various anatomical locations, such as the chest cavity, bladder, or intestines [7]. This case presents the rarest of such occurrences: migration into the PT. A literature review reveals that only 27 cases of PT migration have been reported between 1993 and 2024 [8-10].

The exact pathophysiology of VP catheter migration remains unclear. One hypothesis suggests that during the original surgery, an inadvertent puncture of a major vein, such as the jugular vein, could occur, allowing negative intrathoracic pressure to draw the catheter into the superior vena cava (SVC), right atrium (RA), right ventricle (RV), and eventually the pulmonary artery [11,12]. Another theory posits that erosion of the venous wall by the catheter over time may allow the catheter to enter the vascular system [13]. Migration can occur over varying time frames, from as early as one week to many years postoperatively [14].

Patients with VP shunt catheter migration into the PT can present with a wide range of symptoms, from subtle signs of shunt malfunction, such as headache and nausea, to more severe manifestations like dyspnea and chest pain associated with pulmonary artery thrombosis [15]. In some cases, however, patients may remain asymptomatic or present with nonspecific symptoms, as was the case with our patient, who primarily experienced lower limb edema.

Computed tomography (CT) scans are essential for diagnosing VP shunt migration. Imaging typically shows the course of the catheter, including its abnormal trajectory into the vascular system. In this case, CT revealed the catheter passing through the right internal jugular vein, looping into the SVC, and extending into the PT [5]. Given the potential for life-threatening complications, including heart failure and pulmonary infarction, prompt surgical intervention is necessary [6].

Treatment for VP catheter migration into the PT is highly individualized. The most common approach involves removing the catheter via traction, often under fluoroscopic guidance to prevent damage to the heart valves or the formation of catheter knots [5]. In more complex cases, such as when the catheter becomes entangled or knotted, open cardiac surgery or endovascular retrieval may be necessary [16]. In this case, the catheter was successfully removed through a cervical incision, with fluoroscopic guidance to ensure safe extraction.

## Case presentation

This study presents a rare case of a VP shunt distal catheter migration into the PT in a 17-year-old male patient with hydrocephalus and cerebral palsy. The case was managed at the State Healthcare Institution Morozovskaya City Clinical Hospital of the Moscow Health Department. A retrospective review was conducted, outlining the patient’s clinical presentation, diagnostic imaging findings, surgical treatment, and postoperative outcomes. Additionally, a literature review was performed to contextualize the rarity of the complication and compare management strategies across previously reported cases.

Patient information

The patient, a 17-year-old male, was initially diagnosed with hydrocephalus in infancy and had undergone multiple revisions of his VP shunt system over the years. The most recent shunt revision occurred one year before the current hospital admission. The patient also had comorbidities, including cerebral palsy (Gross Motor Function Classification System Level 5) and structural focal epilepsy in remission. At the time of presentation, the patient’s primary complaint was significant swelling of the lower limbs, which had progressively worsened over the previous 1.5 months. No complaints related to cardiovascular or respiratory function were reported, which made the presentation unusual for a complication involving catheter migration into the pulmonary vasculature.

Clinical examination and initial assessment

Upon admission to the emergency department, a thorough physical examination was performed. The most notable finding was severe edema of the lower extremities, particularly affecting the feet. There were no neurological deficits, and vital signs were within normal limits. The patient’s mother reported no recent fever, respiratory distress, or signs of shunt malfunction, such as headache, nausea, or vomiting.

Given the patient's history of VP shunting and the presence of atypical symptoms, the neurosurgical team conducted an extensive evaluation, starting with routine laboratory tests. A complete blood count revealed mild leukocytosis (11,000/mm³), but biochemical analysis, including liver and renal function tests, showed no significant abnormalities.

Diagnostic imaging

**Figure 1:**
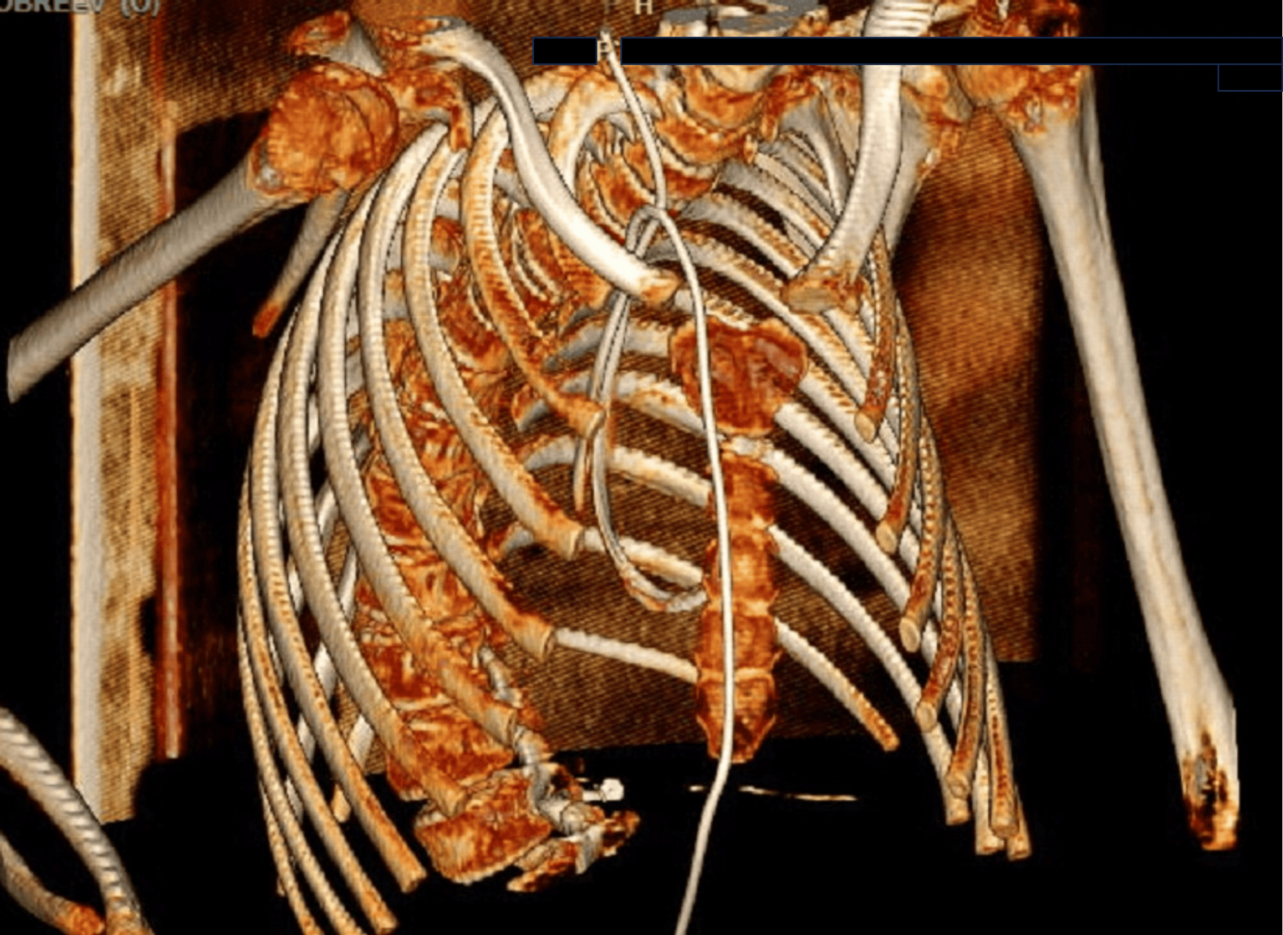
A 3D reconstruction of a chest CT scan showing the migration of the peritoneal catheter into the cardiac cavity through the pulmonary artery.

Considering the progressive nature of the swelling and the history of VP shunting, a CT of the chest was performed. This imaging modality was selected to investigate any potential mechanical or vascular complications associated with the VP shunt system. The CT scan revealed that the distal end of the VP shunt catheter had migrated from the peritoneal cavity into the pulmonary vasculature. Specifically, the catheter was traced through the right internal jugular vein, where it perforated the vessel wall and extended into the SVC, RA, RV, and ultimately into the PT (Figure 1).

The imaging also confirmed that the shunt system was otherwise intact, with no signs of disconnection, valve dysfunction, or CSF leakage. Despite the abnormal location of the catheter within the PT, the patient did not exhibit any cardiovascular symptoms. The CT findings prompted an urgent multidisciplinary discussion regarding the optimal surgical approach for catheter retrieval.

Surgical intervention

Given the severity of the catheter migration and the potential for life-threatening complications such as pulmonary embolism, heart failure, or arrhythmias, a decision was made to proceed with surgical removal of the VP shunt catheter. The operation was planned in collaboration with the cardiovascular surgery team to mitigate risks associated with the procedure.

The patient underwent surgery under general anesthesia. A cervical incision was made along the paraclavicular line on the right side to access the distal catheter, which had migrated into the soft tissues surrounding the jugular vein. Under continuous fluoroscopic guidance, the catheter was carefully extracted from the vascular system through the site of entry at the jugular vein. Fluoroscopy was essential in ensuring that no knots or entanglements formed along the catheter and that the heart valves remained undisturbed during the traction process.

**Figure 2:**
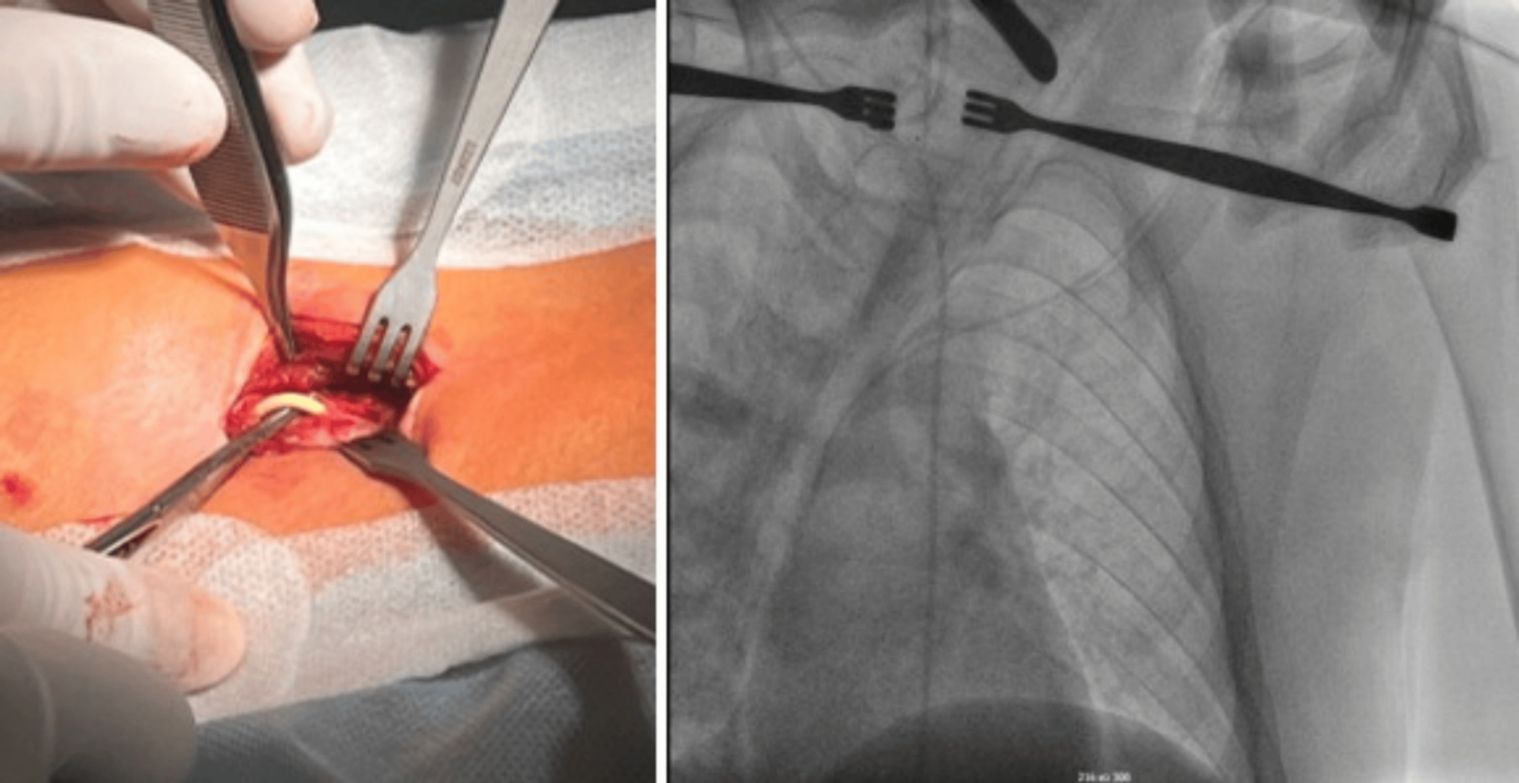
Interoperative photographs A: Incision on the neck and isolation of the peritoneal catheter from the soft tissue; B: Intraoperative X-ray control of catheter removal

Once the distal end of the catheter was safely removed from the PT, the catheter was fully extracted, and the VP shunt system was reimplanted with a new distal catheter tunneled into the peritoneal cavity through a mini-laparotomy in the paraumbilical region, using the patient's previous surgical scar as an entry point. The decision to avoid additional incisions minimized the risk of postoperative complications, such as infection or wound dehiscence (Figure 2).

Postoperative care

Postoperatively, the patient was closely monitored in the intensive care unit for any signs of cardiovascular or pulmonary complications. He received prophylactic antibiotics to reduce the risk of infection, given the invasive nature of the catheter retrieval. Anticoagulant therapy was not deemed necessary, as no thrombotic complications were observed intraoperatively or during the early postoperative period.

The patient exhibited rapid improvement in his symptoms, with a significant reduction in lower limb edema within 24 hours of surgery. He was discharged from the hospital six days postoperatively with instructions for routine follow-up to assess shunt function and monitor for any delayed complications.

## Discussion

Literature review methodology

In addition to the clinical case analysis, a comprehensive literature review was conducted using PubMed, Scopus, Medline, and the Cochrane Library. Keywords such as "ventriculoperitoneal shunt migration," "pulmonary artery," and "hydrocephalus" were used to identify relevant case reports and studies.

Inclusion Criteria

Studies or case reports detailing venous migration of VP shunt catheters, particularly into the pulmonary vasculature or PT; articles published between 1993 and 2024, reports in English, Spanish, and French that provide clinical data on catheter migration; studies focusing on both pediatric and adult patients who experienced venous migration of the VP shunt catheter; studies or reports that used imaging (e.g., CT, MRI, fluoroscopy) to confirm the venous migration and that detail the surgical or endovascular interventions for retrieval; and articles that describe complications, management strategies, and patient outcomes following venous migration of the VP shunt catheter were included in the literature review.

Exclusion Criteria

Studies or reports on ventriculoatrial shunts or cases involving migration to non-venous structures, such as the bladder or gastrointestinal tract; articles focusing on non-venous migratory complications or unrelated mechanical failures of VP shunts; studies where venous migration was not confirmed by imaging or where clinical data on the migration was insufficient; and non-peer-reviewed articles, reviews, or editorials that did not contribute original cases or management outcomes were excluded.

Results

This literature review was conducted according to the Preferred Reporting Items for Systematic Reviews and Meta-Analyses (PRISMA) guidelines, which ensure a structured and transparent approach to reviewing and reporting clinical literature. The PRISMA framework was followed in the identification, screening, eligibility, and inclusion of relevant studies. Our review focused on previously reported cases of VP shunt migration into the pulmonary vasculature, specifically the PT, in pediatric and adult patients.

Search Strategy and Study Selection

A comprehensive search was performed across multiple databases, including PubMed, Scopus, and Google Scholar, using specific search terms: “ventriculoperitoneal shunt migration,” “pulmonary artery,” “hydrocephalus,” and “distal catheter complications.” The search included English, Spanish, and French language studies published between 1993 and 2024. Articles were included if they described cases of VP shunt migration into the PT or pulmonary vasculature, excluding ventriculoatrial shunts and cases of distal catheter migration into other regions, such as the thorax, bladder, or gastrointestinal tract.

**Figure 3:**
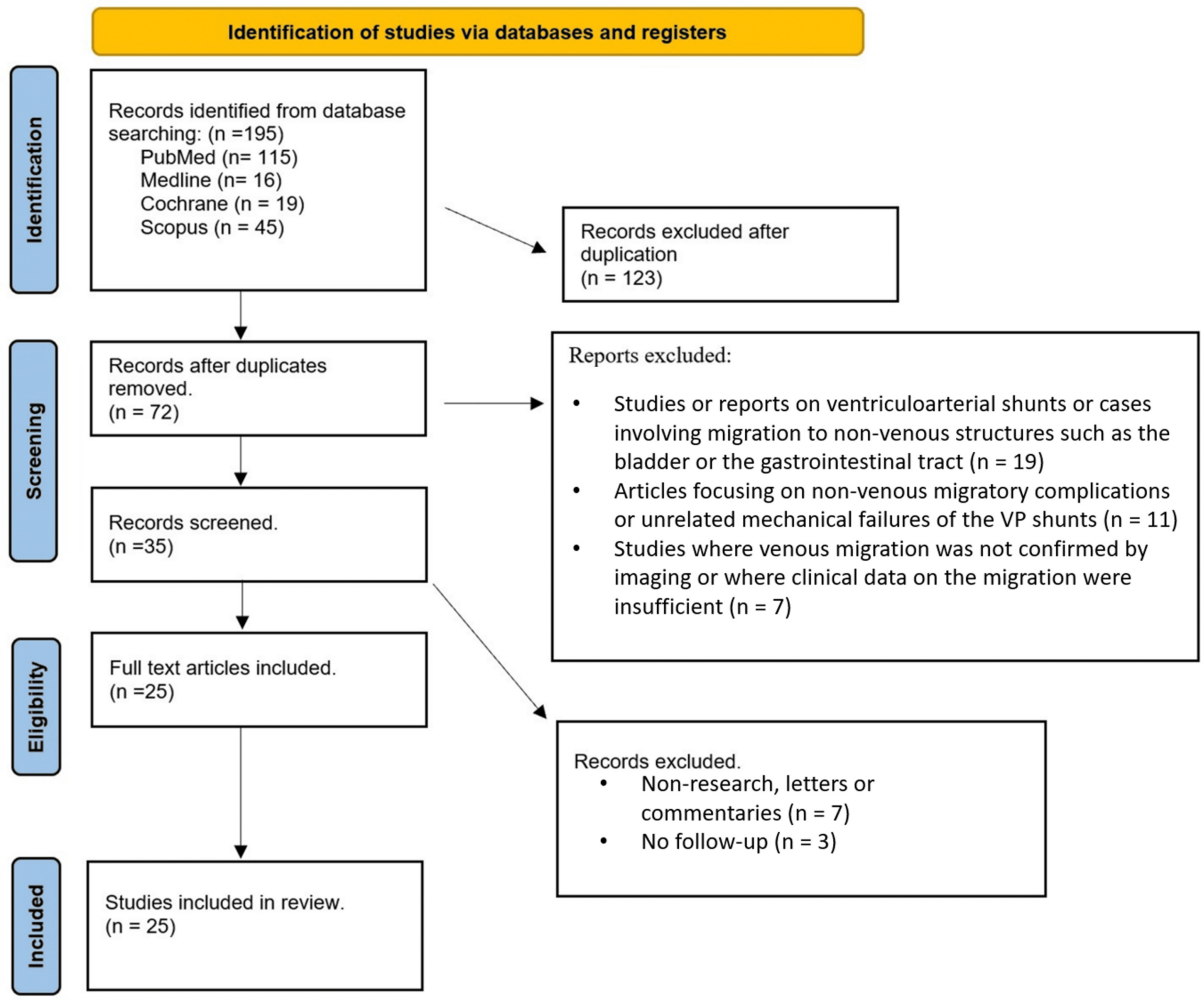
A PRISMA flowchart outlining the study selection process PRISMA: Preferred Reporting Items for Systematic Reviews and Meta-Analyses; VP: ventriculoperitoneal

The initial database search yielded 195 articles. After removing duplicates, 72 articles remained for screening. Titles and abstracts were reviewed for relevance, and 45 articles were excluded based on criteria that did not match the study's focus, such as non-migratory shunt complications or migration to other anatomical areas. The remaining 25 articles were fully reviewed for eligibility. Based on full-text analysis, no additional articles were excluded, and all 25 articles were included in the final review (Figure 3).

Implantation of a ventricular shunt is an effective and common method for treating hydrocephalus [17-19]. Common complications after a ventricular shunt include obstruction, shunt infection, disconnection, shunt dysfunction, pseudocyst formation, and shunt migration [20-21]. Migration of the ventricular shunt into the stomach, gallbladder, urinary bladder, liver, colon, scrotum, chest cavity, and cardiac cavity has been reported. Migration of the catheter into the pulmonary artery is an extremely rare complication of a ventricular shunt; 25 cases were reported in the world literature between 1993 and 2024.

A search of the PubMed database was performed using the terms "migration", "shunt", and "pulmonary artery" in English, Spanish, and French and included cases of catheter migration into the heart and pulmonary vasculature while excluding ventriculoatrial shunts. Twenty-seven cases from 1993 to 2024 were reviewed and analyzed (Table 1).

**Table 1:** A list of publications demonstrating all currently reported cases of distal catheter migration in the pulmonary trunk

Author	Demographic characteristics (country, age)	Time for diagnostics	Cause Of hydrocephalus	Signs and symptoms	Catheter entry point	Catheter installation; It's time to change the catheter	Catheter removal method	Intraoperative complications	Received anticoagulation
Aboukais et al. (2015) [2]	France; 30-year-old	Not specified	Postmeningitic hydrocephalus	Random	Internal jugular vein	Yes, immediately	An incision in the neck area and removal of the catheter	Difficulty removing the catheter using traction (obstruction)	Not specified
Chong et al. (2008) [5]	Korea; 12-year-old	12 months	Hydrocephalus	Asymptomatic	internal jugular vein	Yes, immediately	Retroauricular incision, sternotomy, and pulmonary arteriotomy	Arrhythmias during traction	Not specified
Chong et al. (2008) [5]	Korea; 68-year-old	2 weeks	Subarachnoid hemorrhage	Abdominal pain	Internal jugular vein	Yes, immediately	Cervical incision and transfemoral endovascular removal	Difficulty removing the catheter using traction (obstruction)	Not specified
Dossani et al. (2017) [6]	USA, 30-year-old	1 month	Congenital hydrocephalus	Headache and dizziness	Not specified	Yes, every other day	Retroauricular incision and transfemoral endovascular removal	Difficulty removing the catheter using traction (obstruction)	Not specified
Fewel and Garton (2004) [7]	USA, 16-year-old	1 month	Post-traumatic hydrocephalus	Seizures	External jugular vein	Yes, immediately	Retroauricular incision and catheter removal	No	Not specified
Ghritlaharey (2019) [8]	India, 10-year-old	7 years	Normal pressure hydrocephalus	Headache	Internal jugular vein	Yes, in the abdominal area	Cervical incision and transfemoral endovascular removal	Difficulty removing the catheter using traction (obstruction)	Not specified
Gonzalez et al. (2022) [9]	Spain, 68-year-old	8 years	Post-traumatic hydrocephalus	Asymptomatic	External jugular vein	No, no	The catheter was not removed	No	Yes
Hajdarpasic et al. (2019) [10]	56-year-old	3 years	Obstructive hydrocephalus (meningioma)	Persistent fever due to Klebsiella colonization in a catheter	jugular vein	No, no	An incision in the neck area and removal of the catheter	Not specified	Not specified
Hermann et al. (2009) [11]	Germany, 51-year-old	5 months	Normal pressure hydrocephalus	Random	External jugular vein	Yes, immediately	Cervical incision and transfemoral endovascular removal	Difficulty removing the catheter using traction (obstruction)	Not specified
Imamura and Nomura (2002) [14]	Japan, 76-year-old	18 days	Subarachnoid hemorrhage	Asymptomatic	Internal jugular vein	Yes, in the heart area immediately	An incision in the neck area and removal of the catheter	No	Not specified
Khan et al. (2019) [15]	India, 61-year-old	18 months	Subarachnoid hemorrhage	Sharp chest pain	Jugular vein	Not specified, not specified	Retroauricular incision and catheter removal	The thrombosis was removed by atriotomy	Yes
Kubo et al. (2002) [17]	Japan, 48-year-old	1 month	Subarachnoid hemorrhage	Neck pain	External jugular vein	Yes, immediately	An incision in the neck area and removal of the catheter	No	Not specified
Li et al. (2019) [19]	China, 19-year-old	2 months	Obstructive hydrocephalus (schwannoma)	Gait disturbances	Jugular vein	Yes, immediately	An incision in the cervical region, thoracotomy, and vena cava venotomy	Difficulty removing the catheter using traction (obstruction)	Not specified
Lyon et al. (2016) [20]	USA, 71-year-old	3 weeks	Normal pressure hydrocephalus	Hakim-Adams triad	Not specified	Yes, in 2 months	An incision in the neck area and removal of the catheter	Not specified	Not specified
Moriarty et al. (2019) [22]	USA, 49-year-old	5 years		Shortness of breath during exercise and chest pain	Not specified	Yes, in the heart area, not specified	Transfemoral endovascular removal	No	Not specified
Nguyen et al. (2010) [23]	USA, 28-year-old	8 months	Pseudotumor cerebri	Neck pain	Jugular vein	Yes, not specified	Cervical incision and transfemoral endovascular removal No	No	Not specified
Nordbeck et al. (2010) [24]	Germany, 6-year-old	Not specified	Arachnoid cyst rupture	Fever, shortness of breath, and heart murmur	jugular vein	Yes, not specified	Traction, thoracotomy, and venotomy	Right ventricular insufficiency and arrhythmia	Yes
Patel et al. (2022) [25]	12 year-old	11 years	Not specified	Such as dyspnea, chest pain	jugular vein	No, no	open-heart surgery	No	Not specified
Ralston et al. (2017) [26]	USA, 7-year-old	10 years	Obstructive hydrocephalus (astrocytoma)	Right ventricular failure	internal jugular vein	No, no	An incision in the cervical region, thoracotomy, and venotomy	Difficulty removing the catheter using traction (obstruction)	Not specified
Rodriguez et al. (2023) [27]	Spain, 60-year-old	13 months	Post-traumatic hydrocephalus	Gait disturbances and cognitive problems	internal jugular vein	Yes, immediately	Retroauricular incision and catheter removal	No	Yes
Rodriguez et al. (2023) [27]	Spain, 38-year-old	1 year	Obstructive hydrocephalus (cyst)	Gait disturbance	Jugular vein	Yes, immediately	Cervical incision and transfemoral endovascular removal	Arrhythmias during traction	Yes
Ruggiero et al. (2010) [28]	Italy, 14-year-old	1 month	Not specified	Abdominal pain and irritation of the area behind the ear.	Internal jugular vein	Yes, immediately	An incision in the neck area and removal of the catheter	No	Not specified
Wei et al. (2012) [29]	China, 35-year-old	4 years	Post-traumatic hydrocephalus	Asymptomatic	Internal jugular vein	Yes, in the abdominal area deferred	Retroauricular incision and catheter removal	No	Not specified
Zairi et al. (2012) [30]	France, 63-year-old	1 week	Obstructive hydrocephalus (posterior fossa meningioma)	Random	Internal jugular vein	Yes, not specified	Retroauricular incision and catheter removal	No	Not specified

In the reviewed cases, men predominated (77.78%) in the study groups. Six cases (22.22%) were identified in children; 21 cases (77.78%) were found in adult patients.

The complication rate in ventriculoperitoneal shunting in the pediatric population reached 23% [19]. Risk factors for distal catheter migration in adults include a body mass index (BMI) greater than 30 [20], although no statistical data have been found to indicate that high BMI is a risk factor for ventricular catheter migration in pediatric patients.

One possible mechanism of intravascular migration may be transvenous shunt placement, which is not noticeable during surgery [14]. Imamura and Nomura (2002) [14] believe that after transvenous shunt placement, negative intrathoracic pressure causes the catheter to migrate into the cardiac cavity. Another possible mechanism is catheter-induced erosion of the venous wall since the jugular artery is vulnerable due to its large size, and once the catheter enters the venous system, intrathoracic pressure stimulates its migration into the cardiac cavity [12]. Imamura and Nomura (2002) [14] mention that migration and obstruction usually occur several years after shunting; the time elapsed from the placement of the ventriculoperitoneal shunt system to diagnosis varies from one week to 10 years (Table 1). In our case, the patient's migration occurred within one year.

Patients with migration of the VP shunt catheter into the PT cavity show signs of shunt malfunction: headache, nausea, vomiting, and focal neurological symptoms.

Patients with pulmonary artery thrombosis typically present with dyspnea, chest pain, cough, and symptoms of respiratory failure [21]. Complications such as sepsis or pulmonary embolism may occur [10]. Valve dysfunction was the most common, although it was detected incidentally in 25.9% of cases. In our case, the patient had severe lower limb edema in the absence of other clinical manifestations. The most common treatment for this complication is tractional catheter removal through a cervical or retroauricular incision under fluoroscopy. Patients in whom catheter removal by traction was difficult due to the formation of nodes/adhesions or in the presence of catheter entanglement were treated with endovascular intervention (Table 2). There was one case of an asymptomatic course in a patient in whom the catheter was not removed [10] and one case of endovascular retrieval without a preliminary attempt at mechanical traction retrieval [22]. Chong et al. (2008) [5] indicate that migration of the catheter into the heart can cause arrhythmia, sepsis, heart failure, and pulmonary infarction; therefore, catheter removal is recommended to avoid cardiac complications. In our case, manual traction through an incision on the neck under fluoroscopy control was used; no intraoperative complications or infections were noted while the distal end of the catheter was immersed in the abdominal cavity.

**Table 2:** Treatment methods performed in currently registered cases of distal catheter migration in the pulmonary trunk

Catheter removal method	Number of cases
An incision in the neck area and removal of the catheter	8
Cervical incision and transfemoral endovascular removal	5
Retroauricular incision and catheter removal	4
Traction, thoracotomy, and venotomy	1
The catheter was not removed	1
Retroauricular incision, sternotomy, and pulmonary arteriotomy	1
Retroauricular incision and transfemoral endovascular removal	1
An incision in the cervical region, thoracotomy, and vena cava venotomy	1
Cervical incision and transjugular endovascular removal	1
An incision in the cervical region, thoracotomy, and venotomy	1
Transfemoral endovascular removal	1
Result	25

The most common treatment strategy is neoimplantation of the catheter into the peritoneum, although there is also the possibility of converting the VP shunt into a ventriculo-atrial position or, in rare cases, complete removal of the system [23]. Some patients underwent open thoracotomy due to previous unsuccessful endovascular intervention.

Knotting along the catheter is one of the main intraoperative difficulties identified during traction removal of a migrated catheter. Some patients with the above phenomenon experienced arrhythmias or thrombus formation in the cardiac chambers. Prophylactic anticoagulation was administered in 20% of cases, but due to insufficient information in the published cases, it is not possible to determine whether such prophylaxis was administered to all remaining patients (Table 3). At present, there are no recommendations for anticoagulant therapy in such cases, so in each case, the decision is made collectively with the participation of cardiologists, cardiac surgeons, and hematologists. In our case, the patient did not receive prophylactic anticoagulant therapy.

**Table 3:** Presence of symptoms of a case finding from the total number of registered cases

Signs and symptoms	Number of cases	Percentage
Presence of symptoms	20	80.0%
A chance find	5	20.0%
Result	25	100%

To prevent migration of the distal catheter into the cardiac cavity in adult practice, it is recommended to fix the distal catheter to the peritoneum with a tobacco purse-string suture to prevent upward migration of the catheter [11]. Care must be taken not to damage the wall of the venous vessel in case of profuse bleeding from the neck during tunneling [20]. Direct injury to the jugular vein is rare, but the subcutaneous guidewire should be passed with caution, especially when passing through the supraclavicular fossa [24]. Intraoperative fluoroscopic control is recommended to determine the point of entry of the catheter into the vascular bed and to detect the presence of nodes [2] and is also necessary during catheter removal for real-time visualization, preventing the formation of nodes and entanglement in the heart valves [25]. A multidisciplinary approach is preferable for catheter removal due to the possibility of endovascular intervention or open cardiac surgery [26].

Pathophysiology of Catheter Migration

The exact mechanisms behind VP shunt catheter migration remain speculative, though several theories have been proposed. One prevalent theory is that catheter migration occurs due to accidental puncture of a major vein, such as the internal jugular vein, during initial shunt placement. Imamura and Nomura (2002) hypothesized that negative intrathoracic pressure and venous blood flow can propel the catheter from the SVC into the right atrium, right ventricle, and eventually the PT [14]. This mechanism aligns with the trajectory commonly seen in cases of catheter migration into the pulmonary vasculature.

Additionally, catheter-induced erosion of the venous wall has been identified as a potential cause. Over time, constant mechanical stress exerted by the catheter against the vein wall may lead to erosion, allowing the catheter to enter the vascular system. Li et al. (2019) supported this theory, noting that venous erosion is likely a delayed complication of long-term VP shunt placement, as evidenced by the wide time range for migration, from weeks to years postoperatively [19]. In our case, migration occurred approximately one year after the most recent revision surgery, consistent with this theory.

Risk factors such as BMI, patient age, and surgical technique may also play a role in catheter migration. Khan et al. (2015) found that in adult patients, a higher BMI increases the risk of catheter migration due to mechanical displacement from excess adipose tissue. However, this association has not been definitively established in pediatric cases, indicating that further investigation is needed to fully understand the risk factors for catheter migration in this population [25].

Diagnostic Challenges

Diagnosing VP shunt migration into the PT is often difficult due to the lack of specific symptoms. Many patients, including our own case, present with nonspecific symptoms or are asymptomatic, with the migration discovered incidentally during imaging. For example, Chong et al. (2008) reported that 25.93% of cases were diagnosed incidentally during imaging for unrelated conditions [5]. This underscores the need for regular follow-up imaging in patients with VP shunts, especially when vague symptoms such as swelling or shunt malfunction are present.

Computed tomography is the most reliable imaging modality for diagnosing catheter migration. In a majority of the cases reviewed, CT scans accurately depicted the catheter’s trajectory from the internal jugular vein into the SVC, right atrium, and PT [27]. Fluoroscopy has also been used effectively during surgery to guide catheter removal, particularly when there is a risk of knotting or entanglement. Nguyen et al. (2010) highlighted the importance of fluoroscopic guidance during retrieval to avoid damaging the heart valves or vascular walls [23]. In our case, CT imaging provided a clear roadmap for the surgical team, confirming the catheter’s route through the pulmonary vasculature and allowing for careful planning of the retrieval procedure.

Surgical Management

Surgical intervention is necessary to prevent severe complications such as pulmonary embolism, heart failure, or arrhythmias. The majority of cases are managed through manual traction of the catheter, a technique that is considered safe and effective when performed under fluoroscopic guidance. Fewel and Garton (2004) emphasized that manual traction can usually be performed through a small cervical incision, with fluoroscopy ensuring that no knots or adhesions form along the catheter [7]. In our case, manual traction was successfully employed, with fluoroscopy confirming safe extraction without complications.

In cases where manual traction is not feasible due to catheter entanglement or knotting, endovascular retrieval or open thoracotomy may be required. Dossani et al. (2017) reported successful use of endovascular techniques in 22.2% of cases, especially when the catheter is tightly adhered to the vascular walls [6]. In more extreme cases, such as when the catheter is deeply lodged in the pulmonary artery, open thoracotomy or sternotomy with pulmonary arteriotomy may be necessary, as described by Patel et al. (2022) [25]. These procedures carry higher risks of complications, including infection and prolonged recovery times, but they are sometimes the only option for safely removing the catheter.

In our case, the absence of significant vascular adhesions or knotting allowed for a relatively simple extraction using manual traction. No complications were observed during surgery, and the patient’s symptoms improved rapidly after the procedure (Table 2).

Complications and Outcomes

While the overall prognosis for patients undergoing catheter removal is favorable, complications such as arrhythmias and pulmonary embolism have been reported. Fewer complications occur during manual traction procedures, though arrhythmias have been noted in 14.8% of cases due to irritation of the heart valves during catheter manipulation [28-29]. In contrast, pulmonary embolism is a rarer but more serious complication, typically occurring when the catheter irritates the vascular endothelium, leading to thrombus formation. Hajdarpasic et al. (2019) described a case where a pulmonary embolism developed postoperatively, requiring prompt anticoagulation therapy [10]. While anticoagulation is not routinely recommended in all cases, its use in patients with thrombotic complications should be considered based on individual risk factors [30-31].

The complication of VP shunt migration into the PT, though rare, presents significant clinical challenges, as evidenced by our case and the literature review encompassing 27 similar cases (Table 1). Notably, our study highlights a higher occurrence of this complication among adult patients (77.78%) compared to pediatric patients (22.22%), aligning with the findings. These results underscore the need for vigilance, particularly when managing adult VP shunt cases.

Various factors may contribute to catheter migration, including the patient’s BMI and surgical technique. Our case, similar to others, suggests the possibility of transvenous migration, where negative intrathoracic pressure plays a pivotal role in catheter movement into the pulmonary vasculature. Imamura and Nomura (2002) have previously posited this as a likely mechanism, and its clinical relevance is echoed in the reviewed cases (Table 2).

Clinically, symptoms associated with VP shunt migration into the PT vary widely. While dyspnea, chest pain, and other respiratory symptoms are common in cases involving pulmonary artery thrombosis, 20% of patients were asymptomatic, with migration identified incidentally (Table 3). In our case, the patient presented with lower limb edema, an atypical manifestation of this type of migration. This variability in symptoms necessitates a comprehensive approach to diagnosis, typically with CT imaging, which clearly delineates the catheter’s pathway.

Regarding treatment, manual traction for catheter removal, often under fluoroscopic guidance, was effective in most cases (Table 2). However, more complex cases may require endovascular retrieval or even open thoracotomy if entanglement or adhesions complicate the extraction process. Our case benefited from fluoroscopy during traction, allowing for safe extraction without complications. The outcomes observed reinforce manual traction as an initial approach, reserving more invasive options for cases where simpler techniques prove ineffective [31-32].

Limitations of the study

Retrospective Nature

This single retrospective case and literature review limit definitive conclusions about causality or natural history. Data quality depends on previously reported cases with varying detail and diagnostic methods.

Limited Case Numbers

With only 27 known cases of PT migration, the small sample size hampers robust conclusions and generalizations. Statistical analyses to identify risk factors or outcomes are also limited.

Heterogeneity in Reported Cases

Variations in patient demographics, causes of hydrocephalus, surgical techniques, and clinical outcomes complicate identifying consistent risk factors or patterns. Differences in reporting detail may also influence conclusions.

Lack of Long-Term Follow-Up

Most cases lack extended follow-up, preventing a thorough understanding of complications, recurrence rates, or delayed issues such as shunt malfunction and cardiovascular or neurological events.

Inconsistent Imaging and Diagnostic Criteria

Different diagnostic methods (CT, chest X-ray, echocardiography) were used, potentially affecting accuracy and leading to underreporting or misinterpretation of catheter migration.

Limited Pediatric Data

The majority of cases involve adults, leaving pediatric-specific risk factors and outcomes underexplored due to the small number of pediatric cases.

Publication Bias

Rare, severe, or unusual presentations are more likely to be reported, potentially inflating the perceived prevalence and severity of shunt migration into the PT.

Lack of Standardized Treatment

No uniform protocol exists for managing this rare complication. Surgical and management strategies vary widely, limiting conclusions on the safest and most effective interventions.

## Conclusions

Migration of the distal catheter of the VP shunt is an extremely rare complication; in pediatric practice, only six cases have been registered. Given the small number of described cases, the pathogenesis of this condition has not been sufficiently studied. Migration of the peritoneal catheter into the PT can lead to serious disorders of the cardiovascular system such as heart failure, pulmonary infarction, and severe forms of arrhythmia. A unified treatment strategy has not been developed; however, all publications recommend surgical treatment of such a complication. The choice of surgical tactics is always personalized, taking into account the patient's age, clinical presentation, and radiological picture.

## References

[1] Erkinova Sh B, Babakhanov B Kh, Dzhalalov S Ch (2021). Cost-effectiveness of ventriculoperitoneal shunting versus endoscopic ventriculostomy of the third ventricle in the treatment of hydrocephalus: a literature review. Med Tech.

[2] Aboukais R, Zairi F, Marinho P, Lejeune JP (2015). Management of cardiac migration of a distal shunt catheter: the radiological pitfalls. Neurochirurgie.

[3] Adib SD, Lescan M, Renovanz M (2020). Intracardial catheter migration of a ventriculoperitoneal shunt: pathophysiology and interdisciplinary management. World Neurosurg.

[4] Bullivant KJ, Hader W, Hamilton M (2009). A pediatric experience with endoscopic third ventriculostomy for hydrocephalus. Can J Neurosci Nurs.

[5] Chong JY, Kim JM, Cho DC, Kim CH (2008). Upward migration of distal ventriculoperitoneal shunt catheter into the heart: case report. J Korean Neurosurg Soc.

[6] Dossani RH, Maiti TK, Patra DP, Nanda A, Cuellar H (2017). Endovascular retrieval of migrated distal end of VP shunt from bilateral pulmonary arteries: a technical note. Ann Vasc Surg.

[7] Fewel ME, Garton HJ (2004). Migration of distal ventriculoperitoneal shunt catheter into the heart. Case report and review of the literature. J Neurosurg.

[8] Ghritlaharey RK (2019). Ventriculoperitoneal shunt disconnection, shunt migration, and silent bowel perforation in a 10-year-old boy. J Neurosci Rural Pract.

[9] González-Pombo M, Torri JA, Olivares Blanco M (2023). Ventriculoperitoneal shunt migration into the pulmonary artery: case report and literature review. Neurocirugia (Astur : Engl Ed).

[10] Hajdarpašić E, Džurlić A, Mahmutbegović N, Zahirović S, Ahmetspahić A, Arnautović K, Omerhodžić I (2019). Sepsis caused by bacterial colonization of migrated distal ventriculoperitoneal shunt catheter into the pulmonary artery: a first case report and literature review. World Neurosurg.

[11] Hermann EJ, Zimmermann M, Marquardt G (2009). Ventriculoperitoneal shunt migration into the pulmonary artery. Acta Neurochir (Wien).

[12] Hevia-Rodríguez P, Armendariz-Guezala M, Undabeitia-Huertas J (2023). Distal ventriculoperitoneal shunt catheter migration into the pulmonary artery: a rare complication (Article in Spanish). An Sist Sanit Navar.

[13] Ryugo M, Imagawa H, Nagashima M, Shikata F, Hashimoto N, Kawachi K (2009). Migration of distal ventriculoperitoneal shunt catheter into the pulmonary artery. Ann Vasc Dis.

[14] Imamura H, Nomura M (2002). Migration of ventriculoperitoneal shunt into the heart-case report. Neurol Med Chir (Tokyo).

[15] Khan F, Rehman A, Shamim MS, Bari ME (2015). Factors affecting ventriculoperitoneal shunt survival in adult patients. Surg Neurol Int.

[16] Khan F, Shamim MS, Rehman A, Bari ME (2013). Analysis of factors affecting ventriculoperitoneal shunt survival in pediatric patients. Childs Nerv Syst.

[17] Kubo S, Takimoto H, Takakura S (2002). Peritoneal shunt migration into the pulmonary artery-case report. Neurol Med Chir (Tokyo).

[18] Lancini D, Shetty R (2020). Spontaneous transcardiac migration of a ventriculoperitoneal shunt. Eur Heart J.

[19] Li W, Li Y, Sun Y, Chen L (2019). Migration of a distal ventriculoperitoneal shunt catheter into the pulmonary vasculature: a report of an unusual case and a review of the literature. J Craniofac Surg.

[20] Lyon K, Ban VS, Bedros N, Aoun SG, El Ahmadieh TY, White J (2016). Migration of a ventriculoperitoneal shunt into the pulmonary vasculature: case report, review of the literature, and surgical pearls. World Neurosurg.

[21] Morell RC, Bell WO, Hertz GE, D'Souza V (1994). Migration of a ventriculoperitoneal shunt into the pulmonary artery. J Neurosurg Anesthesiol.

[22] Moriarty JM, Gonzalez Quesada CJ, Wang AC, Yang EH (2019). Knot in the right place. J Vasc Interv Radiol.

[23] Nguyen HS, Turner M, Butty SD, Cohen-Gadol AA (2010). Migration of a distal shunt catheter into the heart and pulmonary artery: report of a case and review of the literature. Childs Nerv Syst.

[24] Nordbeck P, Beer M, Wirbelauer J (2010). Intracardial dislocation of a cranio-peritoneal shunt in a 6-year-old boy. Clin Res Cardiol.

[25] Patel MS, Zhang JK, Khan AS, Alexopoulos G, Khan MQ, Mercier PJ, Kemp JM (2022). Delayed peritoneal shunt catheter migration into the pulmonary artery with indolent thrombosis: a case report and narrative review. Surg Neurol Int.

[26] Ralston A, Johnson A, Ziemer G, Frim DM (2017). Transcardiac migration of ventriculoperitoneal shunt requiring open cardiac surgery: case report and review of the literature. Childs Nerv Syst.

[27] Rodríguez-Sánchez JA, Cabezudo-Artero JM, Porras Estrada LF (2003). Unusual migration of the distal catheter of a ventriculoperitoneal shunt into the heart: case report. Neurosurgery.

[28] Ruggiero C, Spennato P, De Paulis D, Aliberti F, Cinalli G (2010). Intracardiac migration of the distal catheter of ventriculoperitoneal shunt: a case report. Childs Nerv Syst.

[29] Wei Q, Qi S, Peng Y, Fan J, Lu Y (2012). Unusual complications and mechanism: migration of the distal catheter into the heart-report of two cases and review of the literature. Childs Nerv Syst.

[30] Zairi F, du Moulinet d'Hardemaere V, Assaker R (2012). Early cardiac migration of distal shunt catheter. Br J Neurosurg.

[31] Ferras M, McCauley N, Stead T, Ganti L, Desai B (2020). Ventriculoperitoneal shunts in the emergency department: a review. Cureus.

[32] Mpoyi Chérubin T, Augustin K, Jeff N (2024). The role of ventriculocisternostomy in the management of hydrocephalus in Mali and the Democratic Republic of the Congo. Cureus.

